# Quantification of Abdominal Fat in Obese and Healthy Adolescents Using 3 Tesla Magnetic Resonance Imaging and Free Software for Image Analysis

**DOI:** 10.1371/journal.pone.0167625

**Published:** 2017-01-27

**Authors:** Juliana Cristina Eloi, Matias Epifanio, Marília Maia de Gonçalves, Augusto Pellicioli, Patricia Froelich Giora Vieira, Henrique Bregolin Dias, Neide Bruscato, Ricardo Bernardi Soder, João Carlos Batista Santana, Marialena Mouzaki, Matteo Baldisserotto

**Affiliations:** 1 Pediatric Gastroenterologist, Pediatric Gastroenterology Service, Hospital São Lucas da Pontifícia Universidade Católica do Rio Grande do Sul (PUCRS), Porto Alegre, Rio Grande do Sul, Brazil; 2 PUCRS, Porto Alegre, Rio Grande do Sul, Brazil; 3 Veranópolis, Rio Grande do Sul, Brazil; 4 Brain Institute (InsCer), PUCRS, Porto Alegre, Rio Grande do Sul, Brazil; 5 School of Medicine, Universidade Federal do Rio Grande do Sul (UFRGS), Porto Alegre, Rio Grande do Sul, Brazil; 6 Department of Pediatrics, Division of Gastroenterology, Hepatology and Nutrition, The Hospital for Sick Children, Toronto, Canada; 7 Imaging Center Coordinator, Brain Institute (InsCer), PUCRS, Porto Alegre, Rio Grande do Sul, Brazil; Universita degli Studi di Roma La Sapienza, ITALY

## Abstract

**Background and Aims:**

Computed tomography, which uses ionizing radiation and expensive software packages for analysis of scans, can be used to quantify abdominal fat. The objective of this study is to measure abdominal fat with 3T MRI using free software for image analysis and to correlate these findings with anthropometric and laboratory parameters in adolescents.

**Methods:**

This prospective observational study included 24 overweight/obese and 33 healthy adolescents (mean age 16.55 years). All participants underwent abdominal MRI exams. Visceral and subcutaneous fat area and percentage were correlated with anthropometric parameters, lipid profile, glucose metabolism, and insulin resistance. Student’s t test and Mann-Whitney’s test was applied. Pearson’s chi-square test was used to compare proportions. To determine associations Pearson’s linear correlation or Spearman’s correlation were used.

**Results:**

In both groups, waist circumference (WC) was associated with visceral fat area (*P* = 0.001 and *P* = 0.01 respectively), and triglycerides were associated with fat percentage (*P* = 0.046 and *P* = 0.071 respectively). In obese individuals, total cholesterol/HDL ratio was associated with visceral fat area (*P* = 0.03) and percentage (*P* = 0.09), and insulin and HOMA-IR were associated with visceral fat area (*P* = 0.001) and percentage (*P* = 0.005).

**Conclusions:**

3T MRI can provide reliable and good quality images for quantification of visceral and subcutaneous fat by using a free software package. The results demonstrate that WC is a good predictor of visceral fat in obese adolescents and visceral fat area is associated with total cholesterol/HDL ratio, insulin and HOMA-IR.

## Introduction

The prevalence of childhood obesity has become a major public health issue around the world [1,2] Obesity is often associated with metabolic syndrome, which confers an increased risk of cardiovascular events in adulthood [3–5]. Previous studies have shown that central obesity, an indicator of visceral adiposity, is correlated with all the components of metabolic syndrome, namely insulin resistance, dyslipidemia, and hypertension [6,7].

WC (waist circumference) is a good predictor of abdominal adiposity; however, it does not allow for quantification of adipose tissue nor can it distinguish between visceral and subcutaneous fat. The accurate assessment of visceral fat is of utmost clinical importance, given its association with metabolic syndrome components, which in turn, contribute to increased morbidity and mortality. Several techniques are available to measure central adiposity [8–10]. While computed tomography (CT) is the most commonly used imaging modality to measure abdominal fat, Magnetic resonance imaging (MRI) has a similar accuracy [11]. An advantage of MRI is the absence of exposure to ionizing radiation, a limitation that restricts the use of CT in children and adolescents. In addition, the MRI approach to quantifying abdominal adiposity is efficient, allowing for image aqcuisition within 5 minutes.

One aspect that has prevented the use of both CT and MRI is the need for expensive image analysis software in the quantification of abdominal fat [12,13]. However, Irving et al [14] have shown that a free software, NIH Image J, can reliably measure adipose tissue. Even though that study was focused on CT, tone can expect similar results from the analysis of magnetic resonance images obtained using the same software.

Regarding MRI studies of abdominal fat, most have employed equipment with field strength of 1.5 Tesla (T) [11,12,15]. Studies using MRI 3T in adults have shown good accuracy for the quantification of abdominal fat [11]. However, it is important to determine whether 3T MRI has good performance for evaluation of abdominal fat of obese adolescents.

Thus, the aim of this study was to quantify subcutaneous and visceral abdominal fat of adolescents using 3T MRI with the free software and to correlate these findings with anthropometric variables and laboratory parameters that are reflective of metabolic dysregulation.

## Materials and Methods

### Design and Participants

This prospective, cross-sectional study was carried out between March 2013 and October 2014 and included 57 Caucasian adolescents aged 16–18 years. The study was approved the Research Ethics Committee at our university hospital. Sample size was calculated as 23 participants in each group using the PEPI 4.0 software for a significance level of 5%, power of 90%, and a minimum of correlation coefficient 0.5 in BMI association with visceral fat as Kelly et al study.

Exclusion criteria were chronic diseases, hepatorenal disease, and use of hepatotoxic drugs, corticosteroids, or immune suppressants that could promote fat storage in the liver.

The adolescents were randomly selected from a cohort participating in a population study assessing longevity, which was performed in computer system. An informed consent form was signed by all participants or by their guardians. Included subjects were subsequently divided into two groups: Group A included 33 healthy participants and Group B included 24 overweight/obese participants (2 overweight, 22 obese). All underwent anthropometric measurements, laboratory testing, and abdominal MRI measurements.

### Anthropometric Data

Participants were classified according to BMI for age as healthy (Z score ≥ -2 and < 1), overweight (Z score ≥ 1 and < 2), or obese (Z score ≥ 2), following World Health Organization (WHO) guidelines [16]. Participants were weighed wearing light clothes and no shoes, in an upright position, using an anthropometer coupled to a scale (Filizola^®^) certified by the National Institute for Metrology, Quality, and Technology (INMETRO). Body mass index (BMI) was calculated along with Z scores and percentiles, using WHO software AnthroPlus (http://www.who.int/growthref/tools/en/).

WC was measured using non-stretchable plastic tape at the midpoint between the iliac crest and the lowest rib. Waist to height ratio (WHR) was calculated, with a ratio of 0.5 used as cut-point to indicate cardiovascular risk [17,18]. Body surface area was calculated using the DuBois method. [19] Tanner pubertal stage was determined as well [20,21].

### Arterial Pressure

Blood pressure was measured on the day of anthropometric assessment. Two measurements were performed with the subjects in the sitting position after 1 and 5-minute rest periods following their arrival at the medical office. An INMETRO-certified aneroid sphygmomanometer was used. Maximum systolic and diastolic pressures were recorded and categorized according to international guidelines for age, sex, and height as normal, upper limit of normal, and hypertension [22].

### Laboratory Tests

Laboratory tests were performed following a 12-hour fast on the same day of MRI examinations. Lipid profile was determined based on total cholesterol, low-density lipoprotein (LDL) and high-density lipoprotein (HDL) cholesterol, and triglyceride levels using a colorimetric enzymatic method (Mindray-BS 380 Chemistry Analyzer). Fasting glucose was determined using a glucose-oxidase enzymatic method and a Mindray-BS 380 Chemistry Analyzer. Fasting insulin was determined by chemiluminescence. Insulin resistance (IR) was quantified by homeostasis model assessment (HOMA) using the formula: HOMA-IR = fasting insulin (μUI/mL) x fasting glucose (mmol/L)/22.5.

### Abdominal Magnetic Resonance Imaging

All MRI exams were performed at Brain Institute of PUCRS. Images were acquired in a Signa HDxt 3.0T RM scanner (General Electric, Milwaukee, USA) and an eight-element phased array abdominal coil (8-channel coil). Patients were imaged in the supine position and axial T1-weighted fast-spin echo images (FOV 440 mm, matrix 512x512, TR 230, TE 4.40, slice thickness 5.0 mm, gap 1.0 mm, NEX 1) were obtained. Each scan lasted approximately 5 minutes. According to previous studies, a 5 mm thickness slice at the level of L3-L4 discs was selected for the quantification of fat, as it is thought to represent the limit of the upper abdomen and is not influenced by liver or adipose tissue from the buttocks [23,24]. The selected image was saved in .TIFF format.

### Imaging Analysis

**Fig 1:**
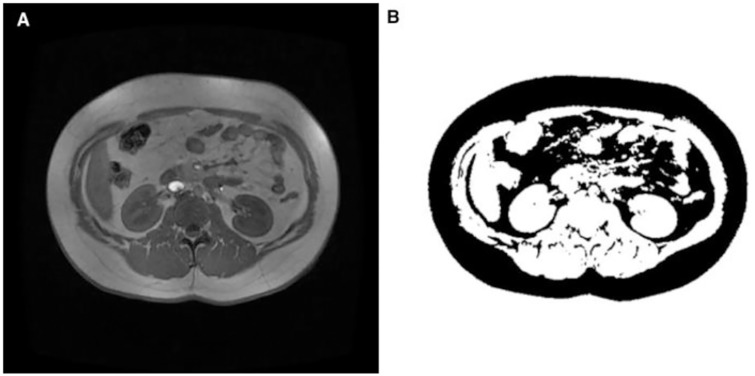
Magnetic resonance image of obese male (BMI 32.59). **A)** .jpg MRI image from L3-L4. **B)** Binary ImageJ image for measurement of fat: fat appears in black. Image shows intra-abdominal (visceral) and subcutaneous fat.

The TIFF images (matrix 512 x 512) were analyzed using ImageJ software (rsbweb.nih.gov/ij) with auto threshold plugin which converts automatically grayscale pixels into binary images, based in a global histogram-derived method. Black pixels represent adipose tissue and white pixels the remaining soft tissue (muscle, solid organs, intestinal loops, and vessels) [25]. Adipose tissue was subsequently categorized into visceral and subcutaneous fat through manual division, which was accomplished by drawing a line following the abdominal wall to separate intra and extra abdominal compartments. Visceral and subcutaneous fat areas (cm^2^) were measured separately (Fig 1) [26].

### Statistical Analysis

Quantitative variables were expressed as means and standard deviation or median and interquartile range. Qualitative variables were expressed as absolute and relative frequencies.

Student’s t test was used to compare group means except in the presence of asymmetric distribution, in which case Mann-Whitney’s test was applied. Pearson’s chi-square test was used to compare proportions. To determine associations between variables in each group, Pearson’s linear correlation (symmetric distribution) or Spearman’s correlation (asymmetric distribution) were used.

The level of significance was set at 5% (*P* ≤ 0.05). All analyses were carried out in SPSS v. 21.0.

## Results

Group A included 16 girls (48.5%) and 17 boys (51.5%), vs. 13 girls (54.2%) and 11 boys (45.8%) in Group B (Table 1). Mean age was 16.8±0.7 and 16.3±0.7 in Groups A and B respectively. There were no significant differences between the groups in terms of gender and age distribution.

**Table 1:** Characteristics of the sample.

Variable (mean ± SD unless indicated)	Healthy (n = 33)	Overweight/obese (n = 24)	*P*
Age (years)	16.8±0.7	16.3±0.7	0.013*
Gender^a^			0.877**
Male	17 (51.5)	11 (45.8)	
Female	16 (48.5)	13 (54.2)	
Systolic pressure (mmHg)	116.9±10.6	121.3±12.8	0.170*
Diastolic pressure (mmHg)	78.7±11.4	84.0±13.1	0.105*
WHR	0.57±0.06	0.44±0.04	< 0.001*
WHR ≥ 0.5^a^	1 (3.0)	22 (91.7)	< 0.001*
Waist circumference (cm)	75.2±6.5	96.4±13.1	< 0.001*
BMI (Z score)	-0.11±0.53	2.45±0.54	< 0.001*
Body surface (m^2^)	1.72±0.16	1.99±0.21	<0.001*

BMI, body mass index; WHR, waist to height ratio.

^a^n (%).

*Student’s t test

**Pearson’s chi-square test.

Healthy: Z score ≥ -2 and < 1; Obese: Z score ≥ 2 (group includes two overweight participants, Z score ≥ 1 and < 2).

WC was significantly higher in Group B (96,4 vs 75,2 cm *P* < 0.001). WHR was also significantly higher in Group B (*P* < 0.001). WHR was higher than 0.5 in only one participant in Group A (3%), vs. 22 (91.7%) participants in Group B. BMI and body surface area were significantly higher in Group B (*P* < 0.001). All study participants were classified as Tanner stage V.

Lipid profile and fasting glucose levels were similar between the two groups. However, fasting insulin and HOMA-IR were higher in group B than Group A (*P* < 0.001). Transaminases and alkaline phosphatase levels were not different between the groups. Elevated transaminase levels (above 22 for grils and 26 for boys) were seen in only one subject in Group B (Table 2).

**Table 2:** Laboratory and MRI findings in healthy and obese adolescents.

Variable (mean ± SD unless indicated)	Healthy (n = 33)	Overweight/obese (n = 24)	*P*
Lipid profile			
Total cholesterol (mg/dL)	159.2±28.0	153.5±31.6	0.478*
HDL cholesterol (mg/dL)	57.9±10.1	50.3±8.0	0.003*
Total cholesterol/HDL ratio	2.84±0.49	3.16±0.52	0.022*
Triglycerides (mg/dL)	70.8±27.4	80.0±44.5	0.339*
Glycemic profile			
Glucose (mg/dL)	80.5±6.1	81.3±7.8	0.653*
Insulin (μUI/mL)^a^	5.1 (4.5–7.0)	9.7 (5.7–12.4)	< 0.001**
HOMA-IR^a^	0.94 (0.79–1.31)	1.73 (1.03–2.16)	< 0.001**
Magnetic resonance imaging			
Total abdominal area (cm^2^)	415±63.5	692±152	< 0.001*
Visceral fat (cm^2^)^a^	16.5 (12.9–21)	57.8 (40–84.5)	< 0.001**
Subcutaneous fat (cm^2^)^a^	54.4 (42–88)	250 (174–347)	< 0.001**
% Abdominal fat (%)	20.9±9.0	44.6±9.5	< 0.001*
% Visceral fat (%)	4.10±1.36	9.11±4.05	<0.001*
% Subcutaneous fat (%)	16.8±8.5	35.4±8.3	<0.001*

HDL, high-density lipoprotein cholesterol; HOMA-IR, homeostatic model assessment—insulin resistance index.

^a^Median (P25 –P75).

*Student’s t test;

**Mann-Whitney’s test.

Healthy: Z score ≥ -2 and < 1; Obese: Z score ≥ 2 (group includes two overweight participants, Z score ≥ 1 and < 2).

As expected, visceral and subcutaneous fat area and percentage, measured by MRI, were significantly higher in Group B (Table 2). Females had higher subcutaneous fat percentage than males in both groups; however, there were no gender differences in terms of visceral fat percentage. Subcutaneous fat area was also higher in girls. Total abdominal area and visceral fat area were significantly higher in Group A boys vs. girls. Boys and girls in Group B differed only in regarding subcutaneous fat percentage, which was higher in girls (*P* = 0.006) (Table 3).

**Table 3:** MRI findings in adolescent boys and girls.

MRI variables (mean ± SD unless indicated)	Healthy (*n* = 33)		*P*	Obese/overweight (*n* = 24)		*P*
	Boys	Girls		Boys	Girls	
Total abdominal area (cm^2^)	450±55.8	379±50.3	0.001	741±166	650±131	0.148*
Visceral fat area (cm^2^)^a^	17 (15–21)	15 (12–19)	0.053	60 (42–95)	56 (38–83)	0.776**
Subcutaneous fat area (cm^2^)^a^	44 (33–55)	80 (54–109)	0.004	219 (146–351)	254 (182–360)	0.424**
% Abdominal fat	16.9±9.4	25.0±6.2	0.007	39.3±9.1	49.1±7.5	0.008*
% Visceral fat	4.10±1.4	4.07±1.3	0.952	8.6±3.4	9.6±4.6	0.545*
% Subcutaneous fat	12.8±8.6	20.9±6.1	0.004	30.7±7.3	39.5±6.9	0.006*

^a^Median (25–75 percentile).

*Student’s t test;

**Mann-Whitney’s test.

Healthy: Z score ≥ -2 and < 1; Obese: Z score ≥ 2 (group includes two overweight participants, Z score ≥ 1 and < 2).

In Group B, both WC and WHR correlated with subcutaneous and visceral fat area (*P* < 0.001 and *P* < 0.01 respectively). In Group A, only WC correlated with visceral fat area (*P* < 0.01); WHR was correlated with subcutaneous fat area in this group (*P* < 0.01). BMI did not correlate with visceral fat (area or percentage) in either group. However, BMI correlated with subcutaneous fat area in Group B (*P* < 0.001) (Table 4) and total abdominal area in both groups (*P* < 0.01 for Group A and *P* < 0.001 for Group B).

**Table 4:** Correlation between anthropometric data and MRI findings.

MRI	Healthy (*n* = 33)				Overweight/obese (n = 24)			
	WC	BMI (Z score)	Body surface	WHR	WC	BMI (Z score)	Body surface	WHR
Total abdominal area (cm^2^)	0.474**	0.451**	0.712***	0.133	0.907***	0.875***	0.791***	0.862***
Visceral fat (cm^2^)	0.456**	-0.067	0.268	0.294	0.426*	0.387	0.326	0.602**
Subcutaneous fat (cm^2^)	0.14	0.344	-0.211	0.455**	0.709***	0.821***	0.490*	0.849***
% Abdominal fat	0.145	0.173	-0.188	0.332	0.287	0.464*	0.031	0.457*
% Visceral fat (%)	0.234	-0.256	0.006	0.205	0.099	0.097	0.018	0.118
% Subcutaneous fat (%)	0.115	0.223	-0.198	0.316	0.282	0.486*	0.027	0.468*

BMI, body mass index; WC, waist circumference; WHR, waist to height ratio.

*P < 0.05;

**P < 0.01;

***P < 0.001

Total/HDL cholesterol ratio was associated with visceral fat area and percentage in Group B, but not in Group A. Similarly, fasting insulin and HOMA-IR were associated with visceral fat area and percentage in Group B. In both groups, there was an association between increased visceral fat percentage and elevated triglyceride levels; however, this reached significance only in Group B (*P* = 0.046) (Table 5).

**Table 5:** Association between metabolic variables and visceral fat.

Variable	Visceral fat area (cm^2^)	% Visceral fat (%)
Total cholesterol/HDL ratio		
Healthy	r = -0.019; *P* = 0.918	r = -0.004; *P* = 0.981
Obese/overweight	r = 0.586; *P* = 0.003	r = 0.522; *P* = 0.009
Insulin levels (μU/mL)		
Healthy	r_s_ = 0.019; *P* = 0.915	r_s_ = 0.051; *P* = 0.780
Obese/overweight	r_s_ = 0.625; *P* = 0.001	r_s_ = 0.553; *P* = 0.005
HOMA		
Healthy	r_s_ = 0.100; *P* = 0.581	r_s_ = 0.065; *P* = 0.720
Obese/overweight	r_s_ = 0.625; *P* = 0.001	r_s_ = 0.556; *P* = 0.005
Triglyceride levels		
Healthy	r_s_ = 0.054; *P* = 0.767	r_s_ = 0.318; *P* = 0.071
Obese/overweight	r_s_ = 0.264; *P* = 0.213	r_s_ = 0.412; *P* = 0.046

HOMA, homeostasis model assessment; r, Pearson correlation coefficient; r_s_, Spearman correlation coefficient.

Healthy: Z score ≥ -2 and < 1; Obese: Z score ≥ 2 (group includes two overweight participants, Z score ≥ 1 and < 2

## Discussion

In this study we were able to show that the use of MRI 3.0 Tesla with the free software package Image J allows for simple, efficient and semi-automatic quantification of abdominal subcutaneous and visceral fat in a cohort of lean and overweight adolescents. Once again it was shown that BMI did not correlate with measures of abdominal adiposity, whereas WC correlated with both visceral and subcutaneous fat tissue. The importance of quantifying visceral fat particularly of overweight and obese subjects was shown again in this study, as visceral adiposity correlated with markers of insulin resistance and dyslipidemia.

To our knowledge there have only been two other studies reporting on the use of MRI 3.0 Tesla in the evaluation of abdominal adipose tissue, both in adults [11,15]. Klopfenstein et al. compared images obtained by MRI 3.0 Tesla to images obtained using CT, which was considered the gold standard [11]. Participants were young adults with a mean BMI of 37 kg/m^2^. This study demonstrated that MRI provides accurate measurements of visceral and subcutaneous adipose tissue. Li et al. reported similar results [15]. In the present study we were able to show that 3T MRI allows clinicians to obtain good quality images in obese adolescents.

The availability of free software Image J suggests that the use of this technology is generalizable. Image J has been previously shown to provide reliable measurements of adipose tissue, with similar accuracy as Slice-O-Matic version 4.3 software (Tomovision) [14]. In addition, Image J features an “eraser” tool that allows for deletion of bowel contents, which can otherwise introduce an overestimate of fat measurements [10,11].

In comparison to other anthropometric parameters, WC correlated best with visceral adiposity area in both groups. Using MRI, Brambilla et al [27]. previously showed that WC is a good predictor of visceral adiposity, whereas BMI predicts subcutaneous adiposity. In our study BMI did not correlate with visceral or subcutaneous fat. Other studies have underscored the superiority of WC to BMI in reflecting visceral adiposity [28–30]. The limitations of using BMI in this clinical setting are numerous. For example, depending on the definition of obesity used, the prevalence of overweight and obesity using the same BMI values can vary widely [31]. Furthermore, it has been shown that BMI fails to identify excess adiposity in over one quarter of children [32], which in turn means that clinicians may fail to identify the need to screen patients at risk for metabolic dysregulation. These data, along with the fact that because of its association with visceral adiposity, an elevated WC is associated with increased future cardiovascular risk, support the inclusion of WC measurements to the routine medical assessment of adolescents.

While WC correlates with markers of abdominal adiposity, it is limited by the fact that it cannot distinguish between visceral and subcutaneous adipose tissue. This is a key distinction when determining the cardiometabolic risk of patients [33]. Access to an efficient and cheap imaging modality, such as the one described in this study, that can distinguish between visceral and subcutaneous fat can, hence, be complementary to the baseline assessment of patients who may be found to have an elevated WC. The additional benefit of this technology is that accurate images can be obtained without the risk of exposing children to ionizing radiation. Laslty, evidence of increasing visceral adiposity can be used as an additional clinical tool to convey to the families the need to be compliant with lifestyle changes aimed at improving their body composition and ultimately decreasing the patients’ future cardiometabolic risk.

We did not observe elevations in the fasting glucose of the adolescents included in this study. However, fasting insulin, HOMA-IR and triglycerides were significantly higher in overweight and obese participants, suggesting the presence of insulin resistance. In addition, HOMA-IR was strongly correlated with visceral adiposity area in obese participants. This finding is in agreement with previously published studies that show a causative relationship between visceral adiposity and insulin resistance [34,35].

In both groups, there was a trend towards increased triglyceride levels and increased visceral fat percentage. It should be noted that only three healthy (9%) and five obese (20.8%) participants had triglycerides above 100 mg/dL. We found a strong correlation of total to HDL cholesterol with visceral fat percentage. This is in accordance with other investigators, who have also shown a strong association between central obesity and dyslipidemia [36,37].

Limitations of the present study include the fact that only Caucasian adolescents of advanced pubertal stage were included. Another limitation is that we did not assess the accuracy of 3T MRI in measuring subcutaneous and visceral abdominal fat but extrapolated data from the adult literature that suggests that this technology is accurate. A study assessing the accuracy of this MRI technology in adolescents would have required exposure to unnecessary radiation, as CT scans are considered the gold standard for these types of measurements.

In conclusion, we show that 3T MRI can provide good quality images using a free software package that allows fast and accurate quantification of visceral and subcutaneous fat in overweight and obese adolescents. The abdominal fat segmentation results demonstrate that WC is a good estimate of visceral and subcutaneous fat and the visceral fat area is associated with total cholesterol / HDL cholesterol, insulin and HOMA-IR.

## Supporting Information

S1 Data (XLSX)

S1 Table Characteristics of the sample. BMI, body mass index; WHR, waist to height ratio. an (%). *Student’s t test **Pearson’s chi-square test. Healthy: Z score ≥ -2 and < 1; Obese: Z score ≥ 2 (group includes two overweight participants, Z score ≥ 1 and < 2).
(DOCX)

S2 Table Laboratory and MRI findings in healthy and obese adolescents. HDL, high-density lipoprotein cholesterol; HOMA-IR, homeostatic model assessment—insulin resistance index. aMedian (P25 –P75). *Student’s t test; **Mann-Whitney’s test. Healthy: Z score ≥ -2 and < 1; Obese: Z score ≥ 2 (group includes two overweight participants, Z score ≥ 1 and < 2).
(DOCX)

S3 Table S3 Title: MRI findings in adolescent boys and girls. aMedian (25–75 percentile). *Student’s t test; **Mann-Whitney’s test. Healthy: Z score ≥ -2 and < 1; Obese: Z score ≥ 2 (group includes two overweight participants, Z score ≥ 1 and < 2).
(DOCX)

S4 Table Correlation between anthropometric data and MRI findings. BMI, body mass index; WC, waist circumference; WHR, waist to height ratio. *P < 0.05; **P < 0.01; ***P < 0.001. Healthy: Z score ≥ -2 and < 1; Obese: Z score ≥ 2 (group includes two overweight participants, Z score ≥ 1 and < 2).
(DOCX)

S5 Table Association between metabolic variables and visceral fat. HOMA, homeostasis model assessment; r, Pearson correlation coefficient; rs, Spearman correlation coefficient. Healthy: Z score ≥ -2 and < 1; Obese: Z score ≥ 2 (group includes two overweight participants, Z score ≥ 1 and < 2.
(DOCX)
