# Supplementary material for: Quantification of Abdominal Fat in Obese and Healthy Adolescents Using 3 Tesla Magnetic Resonance Imaging and Free Software for Image Analysis
Source: PLoS One. 2017 Jan 27;12(1):e0167625. doi: 10.1371/journal.pone.0167625 (PMC5271344; doi:10.1371/journal.pone.0167625)
Supplement: S1 Table — BMI, body mass index; WHR, waist to height ratio. an (%). *Student’s t test **Pearson’s chi-square test. Healthy: Z score ≥ -2 and < 1; Obese: Z score ≥ 2 (group includes two overweight participants, Z score ≥ 1 and < 2). (DOCX) [file pone.0167625.s002.docx]

| **Table 1. Characteristics of the sample** |  |  |  |  |  |  |  |  |
| --- | --- | --- | --- | --- | --- | --- | --- | --- |
| **Variable (mean ± SD unless indicated)** | **Healthy (n = 33)** | **Overweight/obese (n = 24)** | ***P*** |  |  |  |  |  |
| Age (years) | 16.8±0.7 | 16.3±0.7 | 0.013* |  |  |  |  |  |
| Gender^a^ |  |  | 0.877** |  |  |  |  |  |
| Male | 17 (51.5) | 11 (45.8) |  |  |  |  |  |  |
| Female | 16 (48.5) | 13 (54.2) |  |  |  |  |  |  |
| Systolic pressure (mmHg) | 116.9±10.6 | 121.3±12.8 | 0.170* |  |  |  |  |  |
| Diastolic pressure (mmHg) | 78.7±11.4 | 84.0±13.1 | 0.105* |  |  |  |  |  |
| WHR | 0.57±0.06 | 0.44±0.04 | < 0.001* |  |  |  |  |  |
| WHR ≥ 0.5^a^ | 1 (3.0) | 22 (91.7) | < 0.001* |  |  |  |  |  |
| Waist circumference (cm) | 75.2±6.5 | 96.4±13.1 | < 0.001* |  |  |  |  |  |
| BMI (Z score) | -0.11±0.53 | 2.45±0.54 | < 0.001* |  |  |  |  |  |
| Body surface (m^2^) | 1.72±0.16 | 1.99±0.21 | <0.001* |  |  |  |  |  |
| BMI, body mass index; WHR, waist to height ratio. |  |  |  |  |  |  |  |  |
| ^a^n (%). |  |  |  |  |  |  |  |  |
| *Student’s t test **Pearson’s chi-square test. |  |  |  |  |  |  |  |  |
| Healthy: Z score ≥ -2 and < 1; Obese: Z score ≥ 2 (group includes two overweight participants, Z score ≥ 1 and < 2). | | | |  |  |  |  |  |
|  |  |  |  |  |  |  |  |  |
|  |  |  |  |  |  |  |  |  |
