# Supplementary material for: Quantification of Abdominal Fat in Obese and Healthy Adolescents Using 3 Tesla Magnetic Resonance Imaging and Free Software for Image Analysis
Source: PLoS One. 2017 Jan 27;12(1):e0167625. doi: 10.1371/journal.pone.0167625 (PMC5271344; doi:10.1371/journal.pone.0167625)
Supplement: S2 Table — HDL, high-density lipoprotein cholesterol; HOMA-IR, homeostatic model assessment—insulin resistance index. aMedian (P25 –P75). *Student’s t test; **Mann-Whitney’s test. Healthy: Z score ≥ -2 and < 1; Obese: Z score ≥ 2 (group includes two overweight participants, Z score ≥ 1 and < 2). (DOCX) [file pone.0167625.s003.docx]

|  |  |  |  | |  | |  | |  | |  | |  |  |  |  |
| --- | --- | --- | --- | --- | --- | --- | --- | --- | --- | --- | --- | --- | --- | --- | --- | --- |
| **Table 2. Laboratory and MRI findings in healthy and obese adolescents** | | | | | |  | |  | |  | |  | |  |  |  |
| **Variable (mean ± SD unless indicated)** | | | | **Healthy** | | **Overweight/obese** | | ***P*** | |  | |  | |  |  |  |
|  |  |  |  | **(n = 33)** | | **(n = 24)** | |  |  |  | |  | |  |  |  |
| Lipid profile | | | |  | |  | |  | |  | |  | |  |  |  |
| Total cholesterol (mg/dL) | | | | 159.2±28.0 | | 153.5±31.6 | | 0.478* | |  | |  | |  |  |  |
| HDL cholesterol (mg/dL) | | | | 57.9±10.1 | | 50.3±8.0 | | 0.003* | |  | |  | |  |  |  |
| Total cholesterol/HDL ratio | | | | 2.84±0.49 | | 3.16±0.52 | | 0.022* | |  | |  | |  |  |  |
| Triglycerides (mg/dL) | | | | 70.8±27.4 | | 80.0±44.5 | | 0.339* | |  | |  | |  |  |  |
| Glycemic profile | | | |  | |  | |  | |  | |  | |  |  |  |
| Glucose (mg/dL) | | | | 80.5±6.1 | | 81.3±7.8 | | 0.653* | |  | |  | |  |  |  |
| Insulin (µUI/mL)^a^ | | | | 5.1 (4.5 – 7.0) | | 9.7 (5.7 – 12.4) | | < 0.001** | |  | |  | |  |  |  |
| HOMA-IR^a^ | | | | 0.94 (0.79 – 1.31) | | 1.73 (1.03 – 2.16) | | < 0.001** | |  | |  | |  |  |  |
| Magnetic resonance imaging | | | |  | |  | |  | |  | |  | |  |  |  |
| Total abdominal area (cm^2^) | | | | 415±63.5 | | 692±152 | | < 0.001* | |  | |  | |  |  |  |
| Visceral fat (cm^2^)^a^ | | | | 16.5 (12.9 – 21) | | 57.8 (40 – 84.5) | | < 0.001** | |  | |  | |  |  |  |
| Subcutaneous fat (cm^2^)^a^ | | | | 54.4 (42 – 88) | | 250 (174 – 347) | | < 0.001** | |  | |  | |  |  |  |
| % Abdominal fat (%) | | | | 20.9±9.0 | | 44.6±9.5 | | < 0.001* | |  | |  | |  |  |  |
| % Visceral fat (%) | | | | 4.10±1.36 | | 9.11±4.05 | | <0.001* | |  | |  | |  |  |  |
| % Subcutaneous fat (%) | | | | 16.8±8.5 | | 35.4±8.3 | | <0.001* | |  | |  | |  |  |  |
| HDL, high-density lipoprotein cholesterol; HOMA-IR, homeostatic model assessment – insulin resistance index. | | | | | | | | | |  | |  | |  |  |  |
| ^a^Median (P25 – P75). | | | |  | |  | |  | |  | |  | |  |  |  |
| *Student’s t test; **Mann-Whitney’s test. | | | |  | |  | |  | |  | |  | |  |  |  |
| Healthy: Z score ≥ -2 and < 1; Obese: Z score ≥ 2 (group includes two overweight participants, Z score ≥ 1 and < 2). | | | | | | | | | |  | |  | |  |  |  |
