# Supplementary material for: Quantification of Abdominal Fat in Obese and Healthy Adolescents Using 3 Tesla Magnetic Resonance Imaging and Free Software for Image Analysis
Source: PLoS One. 2017 Jan 27;12(1):e0167625. doi: 10.1371/journal.pone.0167625 (PMC5271344; doi:10.1371/journal.pone.0167625)
Supplement: S3 Table — aMedian (25–75 percentile). *Student’s t test; **Mann-Whitney’s test. Healthy: Z score ≥ -2 and < 1; Obese: Z score ≥ 2 (group includes two overweight participants, Z score ≥ 1 and < 2). (DOCX) [file pone.0167625.s004.docx]

| **Table 3.** MRI findings in adolescent boys and girls | |  |  |  |  |  |  |  |
| --- | --- | --- | --- | --- | --- | --- | --- | --- |
|  | **Healthy (*n*= 33)** | |  | **Obese/overweight (*n*= 24)** | |  |  |  |
| **MRI variables** | **Boys** | **Girls** | ***P*** | **Boys** | **Girls** | ***P*** |  |  |
| **(mean ± SD unless indicated)** |  |  |  |  |  |  |  |  |
| Total abdominal area (cm^2^) | 450±55.8 | 379±50.3 | 0.001 | 741±166 | 650±131 | 0.148* |  |  |
| Visceral fat area (cm^2^)^a^ | 17 (15-21) | 15 (12-19) | 0.053 | 60 (42-95) | 56 (38-83) | 0.776** |  |  |
| Subcutaneous fat area (cm^2^)^a^ | 44 (33-55) | 80 (54-109) | 0.004 | 219 (146-351) | 254 (182-360) | 0.424** |  |  |
| % Abdominal fat | 16.9±9.4 | 25.0±6.2 | 0.007 | 39.3±9.1 | 49.1±7.5 | 0.008* |  |  |
| % Visceral fat | 4.10±1.4 | 4.07±1.3 | 0.952 | 8.6±3.4 | 9.6±4.6 | 0.545* |  |  |
| % Subcutaneous fat | 12.8±8.6 | 20.9±6.1 | 0.004 | 30.7±7.3 | 39.5±6.9 | 0.006* |  |  |
| ^a^Median (25-75 percentile). |  |  |  |  |  |  |  |  |
| *Student’s t test; **Mann-Whitney’s test. | |  |  |  |  |  |  |  |
| Healthy: Z score ≥ -2 and < 1; Obese: Z score ≥ 2 (group includes two overweight participants, Z score ≥ 1 and < 2). | | | | | |  |  |  |
|  |  |  |  |  |  |  |  |  |
