# Supplementary material for: Quantification of Abdominal Fat in Obese and Healthy Adolescents Using 3 Tesla Magnetic Resonance Imaging and Free Software for Image Analysis
Source: PLoS One. 2017 Jan 27;12(1):e0167625. doi: 10.1371/journal.pone.0167625 (PMC5271344; doi:10.1371/journal.pone.0167625)
Supplement: S4 Table — BMI, body mass index; WC, waist circumference; WHR, waist to height ratio. *P < 0.05; **P < 0.01; ***P < 0.001. Healthy: Z score ≥ -2 and < 1; Obese: Z score ≥ 2 (group includes two overweight participants, Z score ≥ 1 and < 2). (DOCX) [file pone.0167625.s005.docx]

|  |  |  |  |  |  |  |  |  |  |  |
| --- | --- | --- | --- | --- | --- | --- | --- | --- | --- | --- |
|  |  |  |  |  |  |  |  |  |  |  |
|  |  |  |  |  |  |  |  |  |  |  |
|  |  |  |  |  |  |  |  |  |  |  |
| **Table 4.** Correlation between anthropometric data and MRI findings | | | | |  |  |  |  |  |  |
| **MRI** | | | **Healthy (*n*= 33)** | | | | **Overweight/obese (n = 24)** | | | |
|  |  |  | **WC** | **BMI** | **Body surface** | **WHR** | **WC** | **BMI** | **Body surface** | **WHR** |
|  |  |  |  | **(Z score)** |  |  |  | **(Z score)** |  |  |
| Total abdominal area (cm^2^) | | | 0.474** | 0.451** | 0.712*** | 0.133 | 0.907*** | 0.875*** | 0.791*** | 0.862*** |
| Visceral fat (cm^2^) | | | 0.456** | -0.067 | 0.268 | 0.294 | 0.426* | 0.387 | 0.326 | 0.602** |
| Subcutaneous fat (cm^2^) | | | 0.14 | 0.344 | -0.211 | 0.455** | 0.709*** | 0.821*** | 0.490* | 0.849*** |
| % Abdominal fat | | | 0.145 | 0.173 | -0.188 | 0.332 | 0.287 | 0.464* | 0.031 | 0.457* |
| % Visceral fat (%) | | | 0.234 | -0.256 | 0.006 | 0.205 | 0.099 | 0.097 | 0.018 | 0.118 |
| % Subcutaneous fat (%) | | | 0.115 | 0.223 | -0.198 | 0.316 | 0.282 | 0.486* | 0.027 | 0.468* |
| BMI, body mass index; WC, waist circumference; WHR, waist to height ratio. | | | | |  |  |  |  |  |  |
| **P*< 0.05; ***P* < 0.01; ****P* < 0.001 | | | |  |  |  |  |  |  |  |
