# Supplementary material for: Quantification of Abdominal Fat in Obese and Healthy Adolescents Using 3 Tesla Magnetic Resonance Imaging and Free Software for Image Analysis
Source: PLoS One. 2017 Jan 27;12(1):e0167625. doi: 10.1371/journal.pone.0167625 (PMC5271344; doi:10.1371/journal.pone.0167625)
Supplement: S5 Table — HOMA, homeostasis model assessment; r, Pearson correlation coefficient; rs, Spearman correlation coefficient. Healthy: Z score ≥ -2 and < 1; Obese: Z score ≥ 2 (group includes two overweight participants, Z score ≥ 1 and < 2. (DOCX) [file pone.0167625.s006.docx]

|  |  |  |  |  |
| --- | --- | --- | --- | --- |
| **Table 5.** Association between metabolic variables and visceral fat | | |  |  |
| **Variable** | | | **Visceral fat area (cm^2^)** | **% Visceral fat (%)** |
| Total cholesterol/HDL ratio | | |  |  |
| Healthy | | | r = -0.019; *P* = 0.918 | r = -0.004; *P* = 0.981 |
| Obese/overweight | | | r = 0.586; *P* = 0.003 | r = 0.522; *P* = 0.009 |
| Insulin levels (µU/mL) | | |  |  |
| Healthy | | | r_s_= 0.019; *P* = 0.915 | r_s_= 0.051; *P* = 0.780 |
| Obese/overweight | | | r_s_= 0.625; *P*= 0.001 | r_s_= 0.553; *P* = 0.005 |
| HOMA | | |  |  |
| Healthy | | | r_s_= 0.100; *P* = 0.581 | r_s_= 0.065; *P* = 0.720 |
| Obese/overweight | | | r_s_= 0.625; *P* = 0.001 | r_s_= 0.556; *P* = 0.005 |
| Triglyceride levels | | |  |  |
| Healthy | | | r_s_= 0.054; *P* = 0.767 | r_s_= 0.318; *P* = 0.071 |
| Obese/overweight | | | r_s_= 0.264; *P* = 0.213 | r_s_= 0.412; *P* = 0.046 |
| HOMA, homeostasis model assessment; r, Pearson correlation coefficient; r_s,_ Spearman correlation coefficient. | | | | |
| Healthy: Z score ≥ -2 and < 1; Obese: Z score ≥ 2 (group includes two overweight participants, Z score ≥ 1 and < 2 | | | | |
